# Rediscovered parasitism of *Andrena savignyi* Spinola (Hymenoptera, Andrenidae) by *Stylops* (Strepsiptera, Stylopidae) and revised taxonomic status of the parasite

**DOI:** 10.3897/zookeys.519.6035

**Published:** 2015-09-01

**Authors:** Jakub Straka, Abdulaziz S. Alqarni, Katerina Jůzová, Mohammed A. Hannan, Ismael A. Hinojosa-Díaz, Michael S. Engel

**Affiliations:** 1 Department of Zoology, Charles University in Prague, Viničná 7, CZ-128 44 Praha 2, Czech Republic; 2 Department of Plant Protection, College of Food and Agriculture Sciences, King Saud University, PO Box 2460, Riyadh 11451, Kingdom of Saudi Arabia; 3 Current address: 6-125 Cole Road, Guelph, Ontario N1G 4S8, Canada; 4 Departamento de Zoología, Instituto de Biología, Universidad Nacional Autónoma de México, Mexico City, DF, Mexico; 5 Division of Invertebrate Zoology (Entomology), American Museum of Natural History; Division of Entomology, Natural History Museum, and Department of Ecology and Evolutionary Biology, 1501 Crestline Drive – Suite 140, University of Kansas, Lawrence, Kansas 66045-4415, USA

**Keywords:** Stylopidae, Apoidea, Anthophila, Andrenidae, parasitoid, taxonomy, morphology

## Abstract

Parasitism of Andrena (Suandrena) savignyi Spinola (Hymenoptera: Andrenidae) by *Stylops* Kirby (Strepsiptera: Stylopidae) has been recorded only once, and from an individual collected in Egypt almost a century ago, with the parasite described as *Stylops savignyi* Hofeneder. The recent rediscovery of this *Stylops* from an individual of *Andrena savignyi* permits a reinterpretation of the species and its affinities among other *Stylops*. The bee was collected at flowers of *Zilla spinosa* (Turra) Prantl. (Brassicaceae) in Amariah, Riyadh, Kingdom of Saudi Arabia. Based on DNA barcode sequences from material sampled across Africa, Asia, and Europe, it is apparent that *Stylops savignyi* is conspecific with *Stylops nassonowi* Pierce, and we accordingly synonymize this name (**syn. n.**), with the latter representing the senior and valid name for the species. A differential diagnosis is provided for *Stylops nassonowi* and the morphology of the female is described, as well as the first instars.

## Introduction

Strepsiptera (twisted-wing parasites) are an order of minute entomophagous insects that are found throughout the world. Despite the fact that Strepsiptera comprise relatively few species for a lineage of Holometabola (ca. 600 species), the breadth of hosts is considerable and includes at least seven insect orders (Zygentoma, Blattaria, Mantodea, Orthoptera, Hemiptera, Hymenoptera, and Diptera) (Kathirithamby 2009). There remains considerable debate about their relationship to other holometabolan lineages (e.g., Grimaldi and Engel 2005; Pohl and Beutel 2008, 2013), but most evidence tends to suggest they are near the Coleoptera (e.g., McKenna and Farrell 2010; Ishiwata et al. 2011; Niehuis et al. 2012; Boussau et al. 2014) and some authors in the past have even classified the group as a subordinate among the beetles (e.g., Crowson 1960). The internal phylogeny of Strepsiptera is less controversial in terms of the broader patterns of character transition (e.g., Kinzelbach 1971, 1978, 1990; Pohl 2002; Grimaldi et al. 2005; Pohl and Beutel 2005; Pohl et al. 2005; Bravo et al. 2009; McMahon et al. 2011), although more refined aspects among the ‘higher’ groups and within certain families are in need of revision. There are differences of opinion as to those families recognized, although there are usually 11–13 employed in most summaries of the classification (e.g., Pohl and Beutel 2005; Bravo et al. 2009; Kathirithamby and Engel 2014).

Contributing to the ‘mystery’ of the order is their complex parasitoid life cycle and conspicuous sexual dimorphism, with pronouncedly neotenic females. The male has an ephemeral, free-living adulthood, whereas adult females are obligatory endoparasites, with the sole exception of the basal family Mengenillidae, and are concomitantly tied to their host throughout their maturity (Kathirithamby 1989, 2009; Kinzelbach 1971). In those families more derived than Mengenillidae, adult females have a dramatically reduced body that is largely larviform and is positioned within the host’s body. The more sclerotized cephalothorax of the female extrudes from the host and it is from here that she is able to mate and give birth to her brood. Males seek out parasitized hosts and mate with females who then in turn ultimately produce a large number of free-living first instar larvae, or triungulins (Kathirithamby 2009; O’Connor 1959), that disperse into the surrounding area (Linsley and MacSwain 1957). When the first instars locate a suitable host they attach and eventually invade the body (Kathirithamby 1989, 2009; Kathirithamby et al. 2001). A further complication in the system is found among those first instars of the families Xenidae and Stylopidae which must find a suitable vector that transports them to their new host (Kathirithamby et al. 2012; Linsley and MacSwain 1957). Xenids and stylopids parasitize species of the Euaculeata and they typically position themselves in locations (e.g., among flowers) that will place them into contact with foraging wasps or bees which they can then ride back to the nest and from there invade the brood cells and parasitize the developing immatures (Kinzelbach 1971; Kathirithamby 1989; Pohl and Beutel 2008). Because of this there can at times be disruptions to the developmental process of the host, resulting in noticeable phenotypic alterations (e.g., Smith and Hamm 1914; Salt 1927; Brandenburg 1953; Kathirithamby 1989, 1998; Solulu et al. 1998). Indeed, upon maturity the parasitized host is often sterile or even masculinized such that their ability to collect provisions and provision a new nest is diminished (Smith and Hamm 1914; Salt 1927, 1931), and their behavior altered toward aims other than reproduction (Westwood 1839; Kathirithamby and Hamilton 1995; Hughes et al. 2004; Beani 2006; Linsley and MacSwain 1957; Straka et al. 2011). Accordingly, the newly-emerged first-instar strepsipterans cannot rely on using the host from which they emerged as a vector to a newly established host, and continuing their life cycle requires encounters with new, unparasitized, young individuals (e.g., Kathirithamby et al. 2012). The first instars emerge from their parasitized host on flowers and wait for non-parasitized females of the host species to serve as a vector from the inflorescences to the host’s nest. Within the nest the larvae seek fresh offspring as their final host. Understandably, such larvae are quite mobile, as are all strepsipteran triungulins, and well adapted for concealment and affixation to an appropriate host. For example, first-instar larvae of the genus *Stylops* Kirby have a number of morphological adaptations that provide for a stronger attachment to the host, such as structures on the dorsal and ventral surfaces of their body or enlargement of the pro- and mesotarsi (Pohl 2000; Pohl and Beutel 2004, 2008); however, their behavior on flowers is unknown.

The genus *Stylops* is the most diversified lineage of the family Stylopidae. Species are obligate parasites of solitary bees of the genus *Andrena* Fabricius (Kinzelbach 1971; Jůzová et al. 2015). The taxonomy of species in the genus is problematic, plagued by a plethora of ill-defined epithets established by authors but without defined hypotheses of circumscription for the biological units involved (Straka et al. 2015). In the past, host specificity was often used as the principle guide for species determination, sometimes in the absence of characters intrinsic to the parasite. While host association can be a good guideline, it does not apply universally across all species of *Stylops*. While some species are truly specialists, partial generalists do exist within the genus and these complicate matters for identification (Jůzová et al. 2015). In fact, there are useful morphological details in the first-instar larvae that are of considerable importance in identification and which, in combination with DNA sequences, are also known to reveal various cryptic species (Hayward et al. 2011; Nakase and Kato 2013). Some host-parasite associations are found rarely and for these every newly acquired specimen is an aid toward resolving long-standing taxonomic conundrums, and when suitable field observations are made also further information about possible host specializations, behavior, and ecology. Detailed and modern systematic and biological studies are needed across the order, and numerous hypotheses of species circumscription require critical investigation, with many having remained untested for a century or more.

One such taxonomic mystery that has persisted for nearly a century centers on the proper identity of *Stylops savignyi* Hofeneder (1924). Hofeneder (1924) described his species from two stylopized females of Andrena (Suandrena) savignyi Spinola collected in Egypt, each with one female *Stylops*. Since that time the true identity of this species has represented a persistent problem for the taxonomy of *Stylops*. Here we report the first find of stylopized *Andrena savignyi* from Saudi Arabia, females of which have been found with their stylopid parasite since 1914 (when Hofeneder’s material was collected) and represents a unique opportunity to address the circumscription and identity of *Stylops savignyi*. The species of *Stylops* collected in Saudi Arabia match those described by Hofeneder (1924) and are further identified using new morphological and DNA barcode sequence data. These data reveal the true identity of the parasite species as a new junior synonym of *Stylops nassonowi* Pierce (Pierce 1909) and allow for a modern characterization of the taxon.

## Material and methods

**Figures 1–3.**
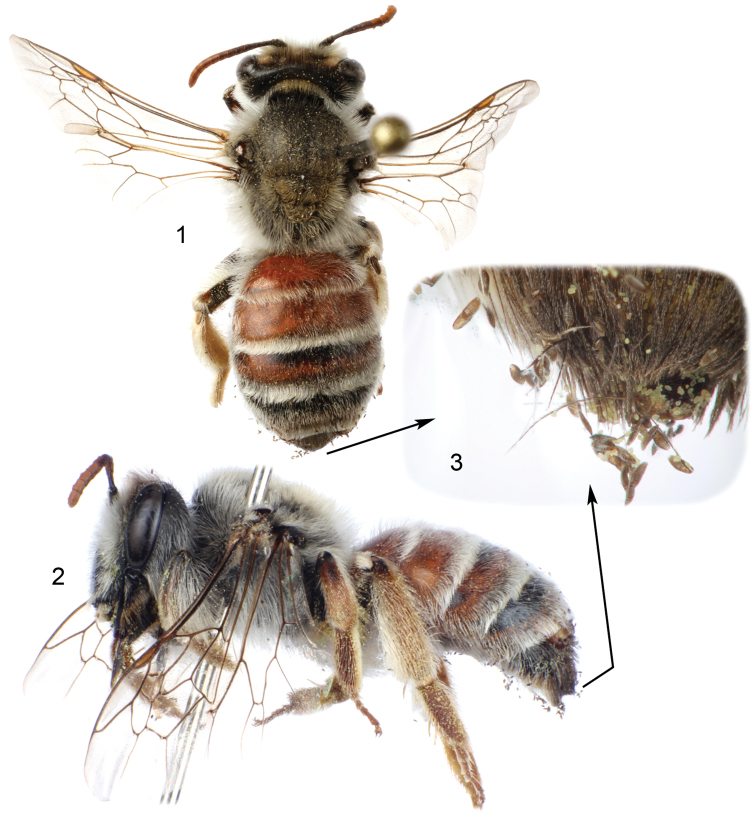
Female of Andrena (Suandrena) savignyi Spinola from Riyadh, Saudi Arabia parasitized by *Stylops nassonowi* Pierce **1** Dorsal habitus of bee **2** Lateral habitus of bee (image inverted); one female of parasite observable at apex of tergum IV **3** Detail of setae at bee’s metasomal apex showing numerous first instars of the parasite.

**Figure 4.**
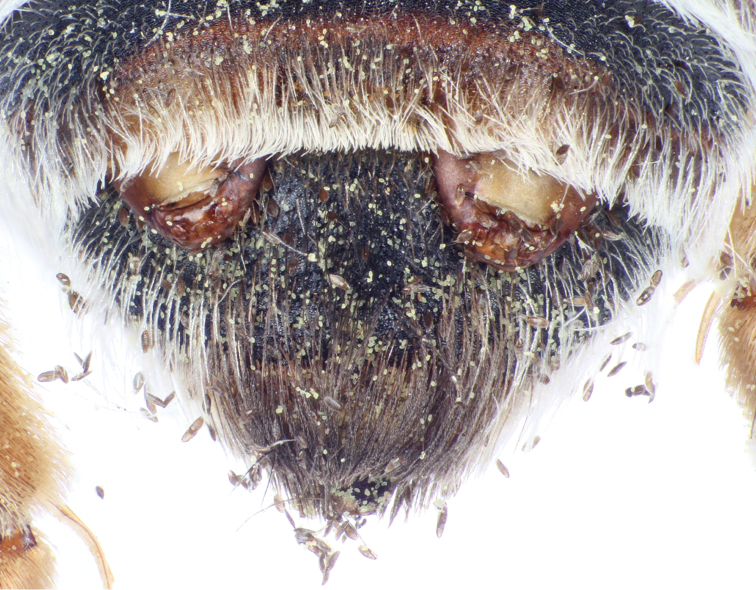
Metasomal apex of female of Andrena (Suandrena) savignyi Spinola from Riyadh, Saudi Arabia parasitized by *Stylops nassonowi* Pierce, and depicting two females of the parasite exposed from under the apex of tergum IV.

Individuals of *Andrena savignyi* were collected mostly from flowers of *Zilla spinosa* (Turra) Prantl. (Brassicaceae) at five localities around Amariah, Riyadh, Saudi Arabia (Al Oyanah, Al Kharj, Rouma, Derab, and Al Amariah, the last of which was where most material was sampled), although the species has also been encountered at various localities throughout Saudi Arabia and the Arabian peninsula (e.g., Dathe 2009; Engel pers. obs.). Details of the collection site are available in Alqarni et al. (2012, 2014a), Engel et al. (2012), and Hannan et al. (2012). At the locality in which the stylopized bee was discovered, general collecting had been underway from September 2010 through September 2012, but all individuals of *Andrena savignyi* were found between 22 February and 10 March 2011 and with peak bee activities at flowers around 20–25 °C. Although there is a diversity flowers around Al Amariah, *Andrena savignyi* was only encountered at *Zilla spinosa* and to a lesser degree at *Rhaphanus sativus* L. and *Eruca sativa* Mill. (both also of Brassicaceae), indicative of its oligolectic pollen-collecting preferences. The stylopized female was collected from *Zilla spinosa*, and she made no attempt to collect pollen from the flowers. The cephalothoraxes of the two female *Stylops* is extruded between the bee’s metasomal terga IV and V (Figs 1, 2), with one on either side of the midline (Fig. 4). Such an orientation is typical for a stylopized bee, where even when parasitized by a single female *Stylops*, the cephalothorax always protrudes from a more lateral position and never from the midline. Measurements of the parasite cephalothoraxes are shown in Table 1.

**Table 1.** Basic measurements of cephalothoraxes of *Stylops aterrimus* Newport and *Stylops nassonowi* Pierce (W = width at spiracles; L = length). All measurements in millimeters.

Species	Voucher	W	L	W of head	L of head	Intermandibular diameter
*Stylops aterrimus*	SAg1	1.35	1.29	0.77	0.34	0.14
	SBm1a	1.29	1.26	0.67	0.31	0.19
	SBm1b	1.34	1.24	0.69	0.29	0.21
	STig2	1.07	1.10	0.64	0.37	0.19
	Ssp1	1.27	1.20	0.67	0.33	0.21
	SCa7	1.31	1.17	0.70	0.31	0.21
	SCa8	1.20	1.17	0.64	0.31	0.19
*Stylops nassonowi*	SCa1	1.41	1.19	0.76	0.3	0.19
	SCa4	1.41	1.27	0.74	0.32	0.19
	SCa5	1.21	1.19	0.70	0.35	0.19
	SCa6	1.09	1.10	0.61	0.31	0.18
	SCa9a	1.19	1.17	0.70	0.32	0.20
	SCa9b	1.33	1.18	0.70	0.30	0.20
	SCa10	1.10	1.11	0.61	0.30	0.18
	SSg1	1.05	1.24	0.60	0.30	0.19
	SHo1	1.36	1.27	0.67	0.34	0.19
	STi2	1.16	1.04	0.70	0.30	0.17
	STi4	1.27	1.19	0.69	0.34	0.19
	STi6	1.26	1.19	0.69	0.31	0.19
	STi5	1.26	1.21	0.73	0.36	0.19

The specimens of *Stylops* examined for the present study (Appendix) were deposited in the King Saud University Museum of Arthropods, Plant Protection Department, College of Food and Agriculture Sciences, King Saud University, Riyadh, Kingdom of Saudi Arabia (KSMA), and the personal collection of Jakub Straka housed at Charles University in Prague, Praha, Czech Republic (JSPC). Material in Saudi Arabia, and from which the new material of stylopized *Andrena savignyi* was sampled, has been collected as part of ongoing bee surveys throughout the country and undertaken by A.S.A., M.A.H., and M.S.E. (e.g., Alqarni et al. 2012a, 2012b, 2013, 2014a, 2014b, 2014c, 2014d; Engel et al. 2012, 2013, 2014; Hannan et al. 2012; Hinojosa-Díaz et al. in press). Those bees with Strepsiptera from other countries were collected into 90–96% ethanol, or with yellow pan-traps and then transferred to ethanol. Individual parasites were removed from dissected bees and subjected to further preparation. Female strepsipterans studied for morphology were cleared using proteinase – a mixture of lysis buffer and proteinase K (Quiagen) heated to 56 °C. The lysis procedure took several hours or overnight. Cleared specimens were cleaned in water several times and then stored in vials with glycerol. Females were observed using an Olympus BX40 light microscope. Temporary slides were prepared with glycerol. First instar larvae were carefully removed from the body of the females and prepared for scanning electron microscopy (SEM) with a JEOL 6380 LV scanning electron microscope. Specimens were dehydrated using progressively more concentrated (90%, 96%, and then 100%) ethanol, each for 5–10 minutes, and then in acetone for 5 minutes. Subsequently, dehydrated samples were critical point dried and coated with gold.

Morphological terminology of first-instar larvae follows that of Pohl (2000), while terminology for females and female puparia follows that of Kinzelbach (1971) and Straka et al. (2014). The following abbreviations were employed: 1L – first-instar larva, F – female, EMP – empty male puparium. The format for the description generally follows that used elsewhere in studies of stylopid systematics (e.g., Straka et al. 2014). Revised descriptions provide a modern framework for species circumscription and build diverse new character sources for studying bee-parasite evolution and systematics (e.g., Engel 2011; Gonzalez et al. 2013), as well as permit the elaboration of patterns of character variation and distribution, reveal relationships, and contribute to a broader understanding of evolution across a clade (e.g., Grimaldi and Engel 2007).

For DNA analysis, the entire body of a female strepsipteran was lysed by Proteinase K (Qiagen). Afterwards, DNA was isolated with a DNA Isolation Kit (Qiagen). Partial DNA sequences were amplified using the primers for Cytochrome oxidase subunit I (COI) (Jůzová et al. in press), and using an annealing temperature of 50 °C. Chromatograms were edited with the program Chromas Lite 2.01 (Technelysium Pty Ltd.) and aligned in BioEdit 7.0.9 (Hall 1999). The online application BLAST was used to reveal any potential contamination in the DNA samples, especially the possibility of amplifying any DNA from the host. Genetic distances were calculated using BioEdit 7.1.3.0 (Hall 1999), under standard computational procedures with the F84 model (Felsenstein 1984).

Distances in DNA base composition were compared pairwise (Table 2). The results show a non-random distribution of genetic distances among individuals in accordance with the published phylogeny of *Stylops* (Jůzová et al. 2015). In the case of material used here, the genetic distance under 2% suggests close relatives. The gap in DNA distance between related individuals within a species and other species is also 2% (1.5–2.5%). Genetic differentiation between the studied populations can be defined according to the present genetic relatedness and the gap.

**Table 2.** DNA distance matrix among samples of *Stylops nassonowi* Pierce, *Stylops aterrimus* Newport, and other representative species. Distances below 2% are highlighted yellow, showing closely related individuals; *Stylops* Kirby from Andrena (Suandrena) savignyi Spinola in Saudi Arabia indicated in red.

*Stylops*		*S. m.*	*S. nev.*	*S. spreta*	*S. m.*	*S. ater*	*S. a.*	*S. a.*	*S. a.*	*S. a.*	*S. a.*	*S. a.*	*S. a.*
	voucher	SFl1	SFu1	SMi1	SNi1	SVa2	SAg1	SBm1	STig1	STig2	Ssp1	SCa7	SCa8
*Stylops nevinsoni*	SFu1	0.1075	-	-	-	-	-	-	-	-	-	-	-
*Stylops spreta*	SMi1	0.1243	0.1456	-	-	-	-	-	-	-	-	-	-
*Stylops melittae*	SNi1	0.0035	0.1081	0.1243	-	-	-	-	-	-	-	-	-
*Stylops ater*	SVa2	0.1440	0.1451	0.1746	0.1453	-	-	-	-	-	-	-	-
*Stylops aterrimus*	SAg1	0.1300	0.1355	0.1511	0.1276	0.1437	-	-	-	-	-	-	-
*Stylops aterrimus*	SBm1	0.1176	0.1353	0.1448	0.1157	0.1337	0.0151	-	-	-	-	-	-
*Stylops aterrimus*	STig1	0.1300	0.1355	0.1511	0.1276	0.1437	0.0000	0.0151	-	-	-	-	-
*Stylops aterrimus*	STig2	0.1410	0.1465	0.1632	0.1383	0.1533	0.0017	0.0178	0.0017	-	-	-	-
*Stylops aterrimus*	Ssp1	0.1300	0.1374	0.1489	0.1276	0.1415	0.0017	0.0134	0.0017	0.0035	-	-	-
*Stylops aterrimus*	SCa7	0.1155	0.1331	0.1426	0.1136	0.1315	0.0168	0.0017	0.0168	0.0196	0.0151	-	-
*Stylops aterrimus*	SCa8	0.1176	0.1353	0.1448	0.1157	0.1337	0.0151	0.0000	0.0151	0.0178	0.0134	0.0017	-
*Stylops nassonowi*	SCa1	0.1286	0.1387	0.1385	0.1245	0.1484	0.0431	0.0414	0.0431	0.0438	0.0412	0.0433	0.0414
*Stylops nassonowi*	SCa2	0.1265	0.1337	0.1375	0.1242	0.1406	0.0410	0.0394	0.0410	0.0416	0.0393	0.0412	0.0394
*Stylops nassonowi*	SCa4	0.1285	0.1357	0.1394	0.1262	0.1426	0.0428	0.0411	0.0428	0.0434	0.0410	0.0429	0.0411
*Stylops nassonowi*	SCa5	0.1345	0.1418	0.1457	0.1321	0.1492	0.0446	0.0429	0.0446	0.0435	0.0427	0.0448	0.0429
*Stylops nassonowi*	SCa6	0.1265	0.1337	0.1375	0.1242	0.1406	0.0410	0.0394	0.0410	0.0416	0.0393	0.0412	0.0394
*Stylops nassonowi*	SCa9	0.1285	0.1357	0.1394	0.1262	0.1426	0.0428	0.0411	0.0428	0.0434	0.0410	0.0429	0.0411
*Stylops nassonowi*	SCa10	0.1330	0.1401	0.1439	0.1305	0.1428	0.0464	0.0447	0.0464	0.0472	0.0446	0.0466	0.0447
*Stylops nassonowi*	SHo1	0.1262	0.1335	0.1392	0.1240	0.1404	0.0375	0.0358	0.0375	0.0378	0.0357	0.0376	0.0358
*Stylops nassonowi*	SSa1	0.1290	0.1340	0.1397	0.1267	0.1410	0.0411	0.0395	0.0411	0.0417	0.0393	0.0413	0.0395
*Stylops nassonowi*	SSg1	0.1285	0.1357	0.1394	0.1262	0.1426	0.0428	0.0411	0.0428	0.0434	0.0410	0.0429	0.0411
*Stylops nassonowi*	STi1	0.1285	0.1357	0.1394	0.1262	0.1426	0.0428	0.0411	0.0428	0.0434	0.0410	0.0429	0.0411
*Stylops nassonowi*	STi2	0.1285	0.1357	0.1394	0.1262	0.1426	0.0428	0.0411	0.0428	0.0434	0.0410	0.0429	0.0411
*Stylops nassonowi*	STi4	0.1285	0.1357	0.1394	0.1262	0.1426	0.0428	0.0411	0.0428	0.0434	0.0410	0.0429	0.0411
*Stylops nassonowi*	STi6	0.1265	0.1337	0.1375	0.1242	0.1406	0.0410	0.0394	0.0410	0.0416	0.0393	0.0412	0.0394

*Stylops*		*S. nass.*	*S. nass.*	*S. nass.*	*S. nass.*	*S. nass.*	*S. nass.*	*S. nass.*	*S. nass.*	*S. nass.*	*S. nass.*	*S. nass.*	*S. nass.*	*S. nass.*
	**voucher**	**SCa1**	**SCa2**	**SCa4**	**SCa5**	**SCa6**	**SCa9**	**SCa10**	**SHo1**	**SSa1**	**SSg1**	**STi1**	**STi2**	**STi4**
*Stylops nevinsoni*	SFu1	-	-	-	-	-	-	-	-	-	-	-	-	-
*Stylops spreta*	SMi1	-	-	-	-	-	-	-	-	-	-	-	-	-
*Stylops melittae*	SNi1	-	-	-	-	-	-	-	-	-	-	-	-	-
*Stylops ater*	SVa2	-	-	-	-	-	-	-	-	-	-	-	-	-
*Stylops aterrimus*	SAg1	-	-	-	-	-	-	-	-	-	-	-	-	-
*Stylops aterrimus*	SBm1	-	-	-	-	-	-	-	-	-	-	-	-	-
*Stylops aterrimus*	STig1	-	-	-	-	-	-	-	-	-	-	-	-	-
*Stylops aterrimus*	STig2	-	-	-	-	-	-	-	-	-	-	-	-	-
*Stylops aterrimus*	Ssp1	-	-	-	-	-	-	-	-	-	-	-	-	-
*Stylops aterrimus*	SCa7	-	-	-	-	-	-	-	-	-	-	-	-	-
*Stylops aterrimus*	SCa8	-	-	-	-	-	-	-	-	-	-	-	-	-
*Stylops nassonowi*	SCa1	-	-	-	-	-	-	-	-	-	-	-	-	-
*Stylops nassonowi*	SCa2	0.0035	-	-	-	-	-	-	-	-	-	-	-	-
*Stylops nassonowi*	SCa4	0.0017	0.0017	-	-	-	-	-	-	-	-	-	-	-
*Stylops nassonowi*	SCa5	0.0018	0.0052	0.0035	-	-	-	-	-	-	-	-	-	-
*Stylops nassonowi*	SCa6	0.0000	0.0033	0.0017	0.0017	-	-	-	-	-	-	-	-	-
*Stylops nassonowi*	SCa9	0.0017	0.0017	0.0000	0.0035	0.0017	-	-	-	-	-	-	-	-
*Stylops nassonowi*	SCa10	0.0053	0.0050	0.0033	0.0070	0.0050	0.0033	-	-	-	-	-	-	-
*Stylops nassonowi*	SHo1	0.0141	0.0134	0.0151	0.0157	0.0134	0.0151	0.0185	-	-	-	-	-	-
*Stylops nassonowi*	SSa1	0.0106	0.0101	0.0118	0.0123	0.0101	0.0118	0.0152	0.0135	-	-	-	-	-
*Stylops nassonowi*	SSg1	0.0017	0.0017	0.0000	0.0035	0.0017	0.0000	0.0033	0.0151	0.0118	-	-	-	-
*Stylops nassonowi*	STi1	0.0017	0.0017	0.0000	0.0035	0.0017	0.0000	0.0033	0.0151	0.0118	0.0000	-	-	-
*Stylops nassonowi*	STi2	0.0017	0.0017	0.0000	0.0035	0.0017	0.0000	0.0033	0.0151	0.0118	0.0000	0.0000	-	-
*Stylops nassonowi*	STi4	0.0017	0.0017	0.0000	0.0035	0.0017	0.0000	0.0033	0.0151	0.0118	0.0000	0.0000	0.0000	-
*Stylops nassonowi*	STi6	0.0000	0.0033	0.0017	0.0017	0.0000	0.0017	0.0050	0.0134	0.0101	0.0017	0.0017	0.0017	0.0017

## Systematics

### Genus *Stylops* Kirby

#### Stylops nassonowi

Taxon classificationAnimaliaStrepsipteraStylopidae

Pierce

Figs 4 , 5 , 6–13 , 20–21 , 22–26

Stylops nassonowi Pierce 1909: 105 [F]. Resurrected name [previously synonymized with *Stylops melittae* Kirby by Kinzelbach (1978)].
Stylops savignyi Hofender 1924: 254 [F]. **New synonyms.**

##### Diagnosis.

Female puparium. The female puparium of *Stylops nassonowi* is almost indistinguishable from its sibling species, *Stylops aterrimus* Newport (compare Figures 6–13, with Figures 14–19). There is probably no stable character that could differentiate female puparia of both species in terms of their morphology and coloration. However, the following few characters occur in one of the species with a higher probability, or are more pronounced in one of the two species: *Stylops nassonowi* has the prothoracic flange of the brood opening typically more produced forward, less numerous mandibular sensilla (less than 10), and pigmentation of the prothorax more uniform except a pale apical part to the abdominal segment of the cephalothoracic venter (well visible in Figures 12, 13). By contrast, *Stylops aterrimus* is more complex in pigmentation than *Stylops nassonowi*, its dark markings on the ventral surfaces of the meso- and metathorax are usually well-developed and the metathorax has a more or less distinct transverse dark band, ultimately giving its apical half a nuanced darker appearance than the basal half (well visible in Figures 14–16). *Stylops nassonowi* differs from other species (such as when compared to *Stylops ater* Reichert, *Stylops melittae* Kirby, *Stylops nevinsoni* Perkins, *Stylops spreta* Perkins, and *Stylops thwaitesi* Perkins) mainly in body and head size (larger than *Stylops nevinsoni*, *Stylops spreta*, and *Stylops thwaitesi*), in the short, dark, basal band (large dark basal band in *Stylops ater*, *Stylops nevinsoni*, *Stylops spreta*, and *Stylops thwaitesi*), described coloration of the cephalothorax, in the shape of the prothoracic flange of the brood opening (strongly curved in *Stylops thwaitesi*; straight in *Stylops spreta*; uniformly curved in *Stylops melittae*, but slightly curved in *Stylops aterrimus* and *Stylops nassonowi*), in the shape of the head corners (strongly curved in *Stylops spreta*, but only slightly curved in the other species), in the shape and sclerotization of the hypostomal and cephalic ridges (strongly sclerotized and dark in *Stylops melittae*, but less pronounced in the other species), and the length of the clypeal sensilla.

First instar. Body elongate as in other species of *Stylops* except for *Stylops melittae*, which has wider abdomen. Head dorsally with two olfactory foveae and four pairs of setae in contrast to *Stylops melittae*, which has seven pairs of setae and no foveae. The frontal margin of the maxillae is not sagging in *Stylops aterrimus* and *Stylops melittae*, in contrast to that of *Stylops nassonowi*. The cervix is indistinct in *Stylops nassonowi* rather than more defined in *Stylops melittae*, the latter possessing a narrower head ventrally. The caudal margins of the dorsal segments have spinullae, except for the pro- and mesothoracic segments, which are covered basally (bases are covered by the tergal margin and therefore not visible rather than fully exposed), while in *Stylops melittae* some spinullae are covered and there is a gap in the center of dorsum where no spinullae are present. The sternal plates are broader than in *Stylops melittae*.

**Figure 5.**
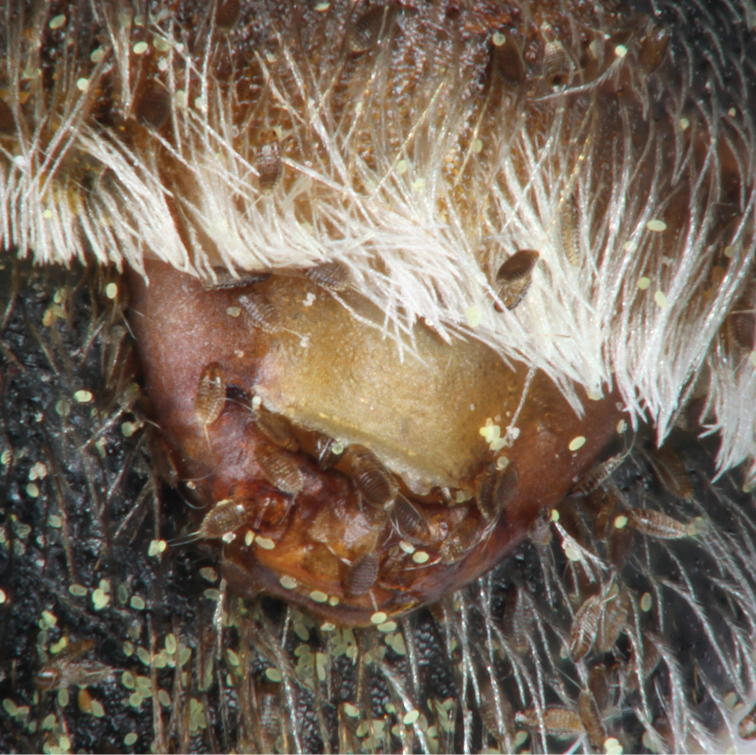
Detail from Figure 4 showing one female of *Stylops nassonowi* Pierce and numerous emergent first instars.

**Figures 6–21.**
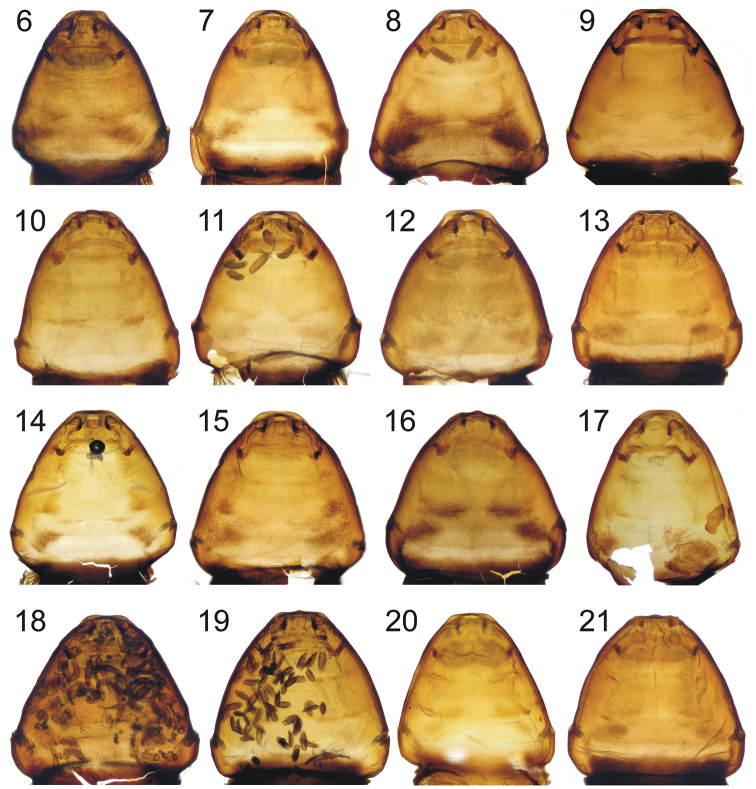
Ventral (6–19) and dorsal views (20, 21) of cephalothoraxes of female puparia from *Stylops nassonowi* Pierce (6–13, 20, 21) and *Stylops aterrimus* Newport (14–19) **6** Voucher SCa5 (Czech Republic) **7** Voucher SCa6 (Czech Republic) **8** Voucher SHo1 (Turkey) **9** Voucher SSa1 (Saudi Arabia) **10** Voucher SSg1 (Czech Republic) **11** Voucher STi2 (Hungary) **12** Voucher STi4 (Czech Republic) **13** Voucher STi6 (Czech Republic) **14** Voucher SAg1 (Tunisia) **15** Voucher SBm1a (Czech Republic) **16** Voucher SBm1b (Czech Republic) **17** Voucher STig2 (Tunisia) **18** Voucher SCa7 (Switzerland) **19** Voucher Ssp1 (Tunisia) **20** Voucher SCa10 (Czech Republic) **21** Voucher STi6 (Czech Republic).

**Figures 22–26.**
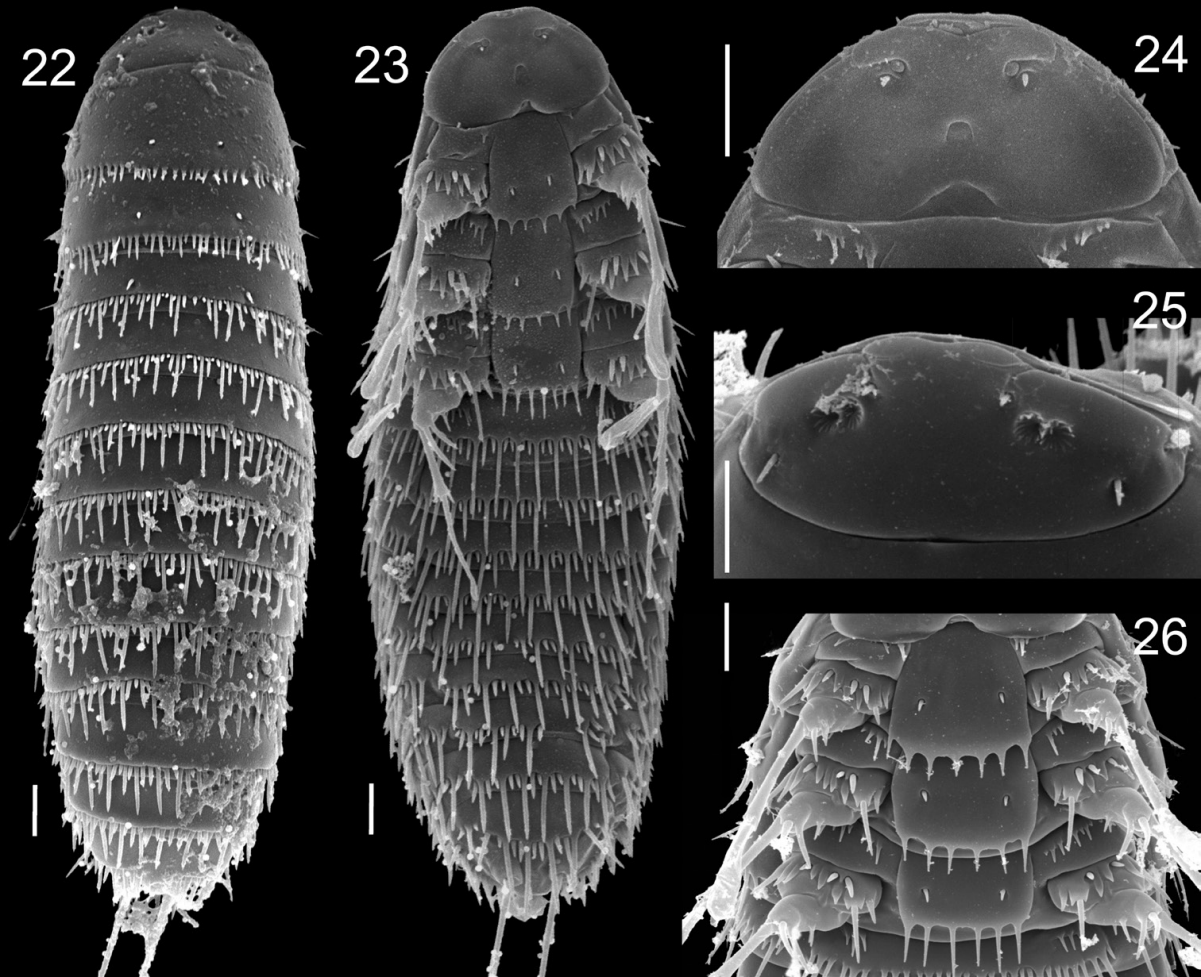
First instar of *Stylops nassonowi* Pierce **22** Dorsal view **23** Ventral view **24** Detail of head, ventral view **25** Detail of head, dorsal view **26** Thoracic segments, ventral view. Scale bars: 10 µm.

##### Redescription.

Female and female puparium. Head two times wider than long, width to length 1.97–2.53 (n = 13, x = 2.15 mm), width 0.60–0.76 mm (x = 0.68 mm), length 0.30–0.36 mm (x = 0.32 mm); head posteriorly defined by single incomplete or ill-defined cephalic ridge on dorsal surface, paired cephalic ridge on ventral surface and posterior head thickening (lower margin of brood opening). Head corners short and narrow on ventral surface, slightly diverging posteriorly, head corners shorter than head on dorsal surface laterally, but inner posterior extension of ventral cephalic ridge (joint of ventral cephalic ridge and posterior head thickening) extends as far as head posterior margin on dorsal surface; ventral cephalic ridge posteromedially oriented; head corners not produced laterally beyond prothorax, head narrower than prothorax and thus cephalothorax continuously diverging posteriorly. Mandibles large, not extending from head contour in ventral view; inner apical tooth well-developed; apex ventrally with 5–8 sensilla, intermandibular distance 0.17–0.20 mm (x = 0.19 mm). Labiomaxillary area about 2–2.5× longer than wide; maxillary area distinctly prominent, overlapping mandible at about one third of its width, maxilla with 7–16 sensilla laterally; labial area without sensilla, more or less prominent and faintly divided into two parts medially (probably postmentum and prementum). Oral ridge (hypopharynx) well developed, rectangular, apically straight, occupying about half of intermandibular area; epipharynx slightly produced, pale, about as long as oral ridge. Hypostomal ridge (from outer margin of mandible to cephalic ridge and separating maxillary area from head corner) slightly sinuous, about as long as intermandibular distance or slightly longer. Labral area well developed, large, arcuate apically, slightly darker than clypeus in most specimens. Clypeus transverse, exceeding mandibles laterally and apically, apex straight or slightly concave, lateral corners prominent, with about 10–30 short sensilla laterally. Brood opening wide, distinctly wider than distance between mandibles; prothoracic flange (dorsal cover of brood opening) sclerotized, arcuate, laterally curved more than medially, apical margin almost straight, in some specimens more produced forward than in others; posterior head thickening (lower margin of brood opening) more uniformly arcuate than flange; overlap of prothoracic flange and posterior head thickening relatively short, about as long as cephalic ridge thick; joint of posterior head thickening and ventral cephalic ridge small, often serrate, slightly lighter than cephalic ridge. Cephalothorax usually slightly wider than long, but longer than wide in some specimens, width to length 0.85–1.18 (x = 1.05), width 1.05–1.41 mm (x = 1.24 mm), length 1.04–1.27 mm (x = 1.18 mm); cephalothorax compact, all segments fused, pigmentation denser laterally than medially. Pro- and mesothoracic intersegmental ridges distinct medially on ventral surface; paired pro- and mesothoracic ridges variable in size, usually distinct on dorsal surface. Pro- and mesothorax uniformly light yellowish-brown except pale prothoracic ridge and slightly darker surrounding integument, posterior part of mesothorax with pair of dark brown spots variable in size (absent in some specimens), distinct lighter area in center of mesothoracic ridge; metathorax uniformly pigmented with paired posterolateral dark brown spots (absent in some specimens); abdominal part of cephalothorax dichromatic, apical part lightest of cephalothorax, nearly transparent, and basal band dark brown, basal band short, not extending toward spiracles, division between basal band and remainder of cephalothorax nearly straight in all parts. Spiracles not prominent, positioned at widest part of posterior part of cephalothorax. Canalis prolifer on abdominal segments I–VII; single median tuba prolifera positioned on posterior third of segments II–VI.

First instar. Body length 135–192 μm (without caudal setae); caudal setae approximately one half body length; with minute terminal leaf-like structure (“Haftlappen”: *vide* Pohl 2000). Head dorsally with four pairs of setae and two olfactory foveae. Mandibles with short setae. Maxillae distinct; frontal margin of maxillae emarginate; rudimentary maxillary palpi circular; ventral opening of praeoral cavity semicircular and isolated from cervix; labium reduced.

Posterior margin of dorsal tergites with spinullae, all spinullae covered basally by tergal margin except for pro- and mesothoracic segments. Each thoracic tergite with two submedian and lateral rows of setae. Coxae broad; each coxa bearing one coxal bristle and 6–7 cuticular outgrowths distributed among three coxal teeth at anterior part of coxa; coxal bristle on pro- and mesothorax at least two times as long as coxal teeth. Trochanterofemur always with femoral spur and bristle almost as long as coxal bristle, and one cuticular outgrowth. Pro- and mesotarsi elongate and slightly enlarged, metatarsi rod-like. Sternal plates broad, with one pair of setae on each plate, with a few outgrowths (about 6) on their posterior margins. Precoxal pleural membrane with small number of microtrichia (about 3) on prothorax, and with transverse row of microtrichia on meso- and metathorax. Short row of cuticular outgrowths (“Spinulaeplatte” *sensu* Borchert 1963) on sternite I. Posterior margins of abdominal sternites with spinullae, some spinullae covered basally. Abdominal segment X with anus, shortened and fused with segment IX, positioned dorsally; segment XI split in two parts and positioned ventrally, bearing caudal setae.

##### DNA sequences.

*Stylops nassonowi* differs significantly in DNA barcode sequence distance, which is consistently about 4% or more from other species, including *Stylops aterrimus*. At the same time, the distances within the species are about 1.5% in distance or even less (Table 2). The only exception is an individual collected in eastern Turkey, which differs from all other sequenced individuals of *Stylops nassonowi* in 1.3–1.9% distance and might represent an isolated population or perhaps different subspecies. Greater sampling is needed across the distribution of the species, particularly the Levant and elsewhere in Arabia.

## Discussion

Pierce (1909) described *Stylops nassonowi* based on a figure provided by Nikolai V. Nasonov (1855–1939) in a comparative morphological study of material the latter ascribed to *Stylops melittae* and had taken from a female of Andrena (Plastandrena) pilipes Fabricius (Nasonov 1893a, 1893b). In establishing his new species, Pierce (1909) listed both Germany and Egypt as comprising type localities [referring to the host as *Andrena carbonaria* (Linnaeus), often considered the senior synonym for *Andrena pilipes*]; however, no specific locality is mentioned by Nasonov (1893a, 1893b), who could have had material from various places across the Palaearctic. At the time Pierce was publishing, available records of stylopized *Andrena pilipes* and ascribed by Pierce (1909) to *Stylops nassonowi* were known from Egypt (Saunders 1872), France (Pérez 1886), and Germany (Friese 1891), and it was from the former and the latter that he likely based his designation. Given this, we consider the type locality to be uncertain and clarification will rely on the eventual designation of a neotype as Nasonov’s material is apparently no longer extant. We have hesitated from designating a neotype herein as further investigation into the ultimate disposition and survival of Nasonov’s collection is needed.

Phylogenetic analysis of species of *Stylops* sampled from a diversity of hosts (Jůzová et al. 2015) coupled with the new DNA barcode sequences of the present study further demonstrate that the *Stylops* collected in Saudi Arabia belong to the species complex consisting of *Stylops aterrimus* and *Stylops nassonowi*. From the results we are able to define an eastern lineage, the oldest available name of which is *Stylops nassonowi* and a western lineage which accords with *Stylops aterrimus*. These results further establish the synonymy of *Stylops savignyi* from *Andrena savignyi* as a synonym of *Stylops nassonowi*, and the species appears to be a partial generalist, victimizing multiple species in separate subgenera of *Andrena* (*Plastandrena* Hedicke and *Suandrena* Warncke) (Appendix).

*Stylops aterrimus* and *Stylops nassonowi* are close sibling species and are almost indistinguishable morphologically. The two lineages exhibit sequence distances of about 4%, which is quite distinct when compared to many other species. Although we readily admit that there is no definable metric value of percent sequence difference for conferring specific status, 4% is greater than many other closely related species that are easily diagnosed on the basis of additional characters outside of the sequences themselves. Intraspecific variance in the DNA distances of each species is well below 2% and the variability is not overlapping (Table 2), further suggestive of individual evolutionary lineages. Both of these species are more than 10% distant from other common species of *Stylops* in terms of their DNA barcode sequence (Table 2: Jůzová et al. 2015). *Stylops aterrimus* and *Stylops nassonowi* seem to be largely allopatric across Europe, with their place of contact around the Czech Republic, where both species were recorded although not necessarily from precisely the same locality within that country. The border of contact between the two species is, of course, expected in other countries through Central Europe as well as in northern Africa. This split into a western and eastern species is perhaps a reflection of Pleistocene glaciation across Europe during the Pleistocene, as areas such as western France and Spain were spared from extensive ice coverage, while the same was true for the Italian Peninsula and Balkans, with some narrow corridors of contact north of the Alps (Ehlers and Gibbard 2004; Ehlers et al. 2011). Naturally, such a pattern of distribution and contact requires further testing through the acquisition of considerably more material, and finer-scale phylogeographic study, ideally coupled with some degree of calibration for purposes of dating. For the moment our limitations largely reflect the infrequent collection of strepsipterans, particularly as many entomologists ignore the presence of such parasites.

The present study demonstrates how a seemingly happenstance and serendipitous encounter with a stylopized female of *Andrena savignyi* permitted a significant shift in a long-standing taxonomic obstacle. Clarification of the identity of *Stylops savignyi* provides one further step toward a revised classification of *Stylops* supported by both morphological and molecular data. Given the increased awareness of native pollinators (many of which are wild bees) and their importance for ecosystem health, numerous initiatives are underway to study such species. These endeavors are making available new samples from previously under-collected regions and with this increased effort the probability of acquiring fresh material of their parasites, some unseen for decades. Melittologists and pollination biologists should develop an awareness and maintain alertness for stylopized females, and where possible obtain data on their impact on the host’s behavior and development as it not only makes less known the Strepsiptera but simultaneously enhances our knowledge of the hosts.

## Supplementary Material

XML Treatment for Stylops nassonowi

## Appendix

**Material of species of *Stylops* used for taxonomic comparison**

Here we provide specimen and collective event details for the various specimens of species of *Stylops* used in our comparative studies, along with the hosts from which they were sampled. In addition, specimens tied to specific sequences deposited in GenBank are identified and their numbers provided.

### *Stylops ater* Reichert

*Stylops ater* Reichert 1914: 151 [♂]. Type locality: Merseburg, Germany.

**Material examined. Czech Republic**: Bohemia: Prokopské údolí, Praha-Jinonice, 1F, host: Andrena (Melandrena) vaga Panzer, 1♂, 13.iii.2007, J. Straka lgt., voucher SVa2, DNA barcode, GenBank: KF803529.

### *Stylops aterrimus* Newport

Figs 14–19

*Stylops spencii* auctorum (*nec* Pickering 1836).

*Stylops aterrimus* Newport 1851: 340 [♂]. Type locality: Hampstead, Great Britain.

**Material examined. Czech Republic**: Bohemia, Velký Luh, sandpit, 2FF+1EMP, host: Andrena (Plastandrena) bimaculata (Kirby), 1♀, 20.iv.2010, J. Straka lgt., voucher SBm1, DNA barcode, GenBank: KP213298. **Switzerland**: Zürich env., 1F, host: Andrena (Hoplandrena) carantonica Pérez, 1F, 25.v.2010, collector unknown, voucher SCa7, DNA barcode, GenBank: KP213300; ditto, 1F+1EMP, voucher SCa8, DNA barcode, GenBank: KP213299. **Tunisia**: Gafsa env., 1F, host: Andrena (Plastandrena) bimaculata, 1F, 1.iv.2006, J. Batelka et J. Straka lgt., voucher STig2, DNA barcode, GenBank: KF803522; Tamerza env., 1F, host: Andrena (Agandrena) agilissima (Scopoli), 1F, 31.iii.2006, J. Batelka et J. Straka lgt., voucher SAg1, DNA barcode, GenBank: KF803428; ditto, 1F, host: Andrena (Plastandrena) bimaculata, 1F, voucher STig1, DNA barcode, GenBank: KF803521; Wadi Raml, 4.5 km E Douz, 1F, host: Andrena (Hoplandrena) sp., 1F, 4.iv.2006, J. Batelka et J. Straka lgt., voucher Ssp1, DNA barcode, GenBank: KF803504.

### *Stylops melittae* Kirby

*Stylops melittae* Kirby 1802: 113 [♂]. Type locality: not indicated.

**Material examined. Czech Republic**: Bohemia, Čelákovice env., 1F, host: Andrena (Zonandrena) flavipes Panzer, 1♂, 1.v.2006, J. Batelka lgt., voucher SFl1, DNA barcode, GenBank: KF803453; Bohemia, Prokopské údolí, Praha-Jinonice, 1F, host: Andrena (Melandrena) nigroaenea (Kirby), 1♀, 6.iv.2009, J. Straka lgt., voucher SNi16, DNA barcode, GenBank: KF803488.

### *Stylops nassonowi* Pierce

Figs 4–13, 20–26

*Stylops nassonowi* Pierce 1909: 105 [F]. Type locality: ‘Egypt and Germany’ (*vide* Discussion).

*Stylops savignyi* Hofender 1924: 254 [F]. Type locality: Egypt.

**Material examined. Czech Republic**: Bohemia, Divoká Šárka, Praha-Liboc, 1F, host: Andrena (Hoplandrena) carantonica Pérez, 1♂, 15.iv.2006, J. Straka lgt., voucher SCa2, DNA barcode, GenBank: KF803434; Bohemia, Chvalské skály, Praha-Horní Počernice, 2FF, host: Andrena (Hoplandrena) carantonica, 1♀, 3.vi.2005, J. Straka lgt., voucher SCa9, DNA barcode, GenBank: KF803436; Bohemia, Sušice env., 1F, host: Andrena (Plastandrena) tibialis (Kirby), 1♀, 9.iv.2007, L. Dvořák lgt., voucher STi1, DNA barcode, GenBank: KF803518; ditto, 2FF, voucher STi6, DNA barcode, GenBank: KP213303; Bohemia, Závišín, Blatná env., 1F, host: Andrena (Plastandrena) tibialis, 1♂, 4.iv.2009, P. Bogusch lgt., voucher STi4, DNA barcode, GenBank: KP213302; Moravia, Dolní Dunajovice env., 1F+1EMP, 16.iv.2007, P. Bogusch lgt., voucher SCa10, DNA barcode, GenBank: KP213304; Moravia, Dolní Věstonice env., 1F, host: Andrena (Hoplandrena) carantonica, 1♂, 5.iv.2008, J. Batelka et J. Straka lgt., voucher SCa5, DNA barcode, GenBank: KP213305; Moravia, Dolní Věstonice env., 1F+1EMP, host: Andrena (Hoplandrena) carantonica, 1♂, 6.iv.2009, P. Bogusch lgt., voucher SCa6, DNA barcode, GenBank: KF803435; ditto, 3FF, host: Andrena (Hoplandrena) spinigera (Kirby), 1♂, voucher SSg1, DNA barcode, GenBank: KF803503; Moravia, Lednice env., 1F, host: Andrena (Hoplandrena) carantonica, 1♀, 13.vi.2006, J. Straka lgt., voucher SCa1, DNA barcode, GenBank: KF803433; **Hungary**: Budaörs, Budapest env., 1F, host: Andrena (Hoplandrena) carantonica, 1♀, 25.iv.2009, J. Straka et P. Bogusch lgt., voucher SCa4, DNA barcode, GenBank: KP213301; Örkeny (puszta), 1F, host: Andrena (Plastandrena) tibialis, 1♂, 24.iv.2009, J. Straka et P. Bogusch lgt., voucher STi2, DNA barcode, GenBank: KF803519; **Saudi Arabia**: Riyadh, Al Amariah, Majra Al-gasim, 2FF, host: Andrena (Suandrena) savignyi Spinola, 1♀, 5.iii.2011, M.A. Hannan lgt., voucher SSa1, DNA barcode, GenBank: KP213306; **Turkey**: Hakkari prov., Gözeldere 25 km E, 1F, host: Andrena (Plastandrena) sp., 1♀, 22.vi.2010, Mi. Halada lgt., voucher SHo1, DNA barcode, GenBank: KF803463.

### *Stylops nevinsoni* Perkins

*Stylops nevinsoni* Perkins 1918: 71 [F]. Type locality: Great Britain.

**Material examined. Czech Republic**: Bohemia, Chýnice, 1F, host: Andrena (Andrena) fulva (Müller), 1♀, 22.iv.2006, J. Batelka et J. Straka lgt., voucher SFu1, DNA barcode, GenBank: KF803457.

### *Stylops spencei* Pickering

*Stylops spencei* Pickering 1836: 168 [F]. Type locality: Great Britain.

**Material examined. Czech Republic**: Bohemia, Chýnice, 1F, host: Andrena (Micrandrena) minutula (Kirby), 1♀, 22.iv.2006, J. Batelka et J. Straka lgt., voucher SMi1, DNA barcode, GenBank: KF803477.

### *Stylops thwaitesi* Perkins

*Stylops thwaitesi* Perkins 1918: 70 [♂, F]. Type locality: Great Britain.

**Material examined. Spain**: Maranchón 3km NW, Castilla-La Mancha prov., 1F, host: Andrena (Taeniandrena) albofasciata Thomson, 1♀, 10.iv.2012, K. Černá, K. Jůzová et J. Straka lgt., voucher SOv3, DNA barcode, GenBank: KF803494.
